# Tracing the evolution of fatty acid‐binding proteins (FABPs) in organisms with a heterogeneous fat distribution

**DOI:** 10.1002/2211-5463.12840

**Published:** 2020-03-31

**Authors:** Yuru Zhang, Junmei Zhang, Yanhua Ren, Ronghua Lu, Liping Yang, Guoxing Nie

**Affiliations:** ^1^ College of Fisheries Henan Normal University Xinxiang China; ^2^ College of Fisheries Engineering Technology Research Center of Henan Province for Aquatic Animal Cultivation Henan Normal University Xinxiang China

**Keywords:** evolution, FABPs, fat distribution, fatty acid‐binding proteins, gene structure

## Abstract

The distribution of fat among both invertebrate and vertebrate groups is heterogeneous. Studies have shown that fatty acid‐binding proteins (FABPs), which mainly bind and transport fatty acids, play important roles in the regulation of fat storage and distribution. However, the systematic and genome‐wide investigation of *FABP* genes in organisms with a heterogeneous fat distribution remains in its infancy. The availability of the complete genomes of *Caenorhabditis* *elegans*, *Callorhinchus* *milii*, and other organisms with a heterogeneous fat distribution allowed us to systematically investigate the gene structure and phylogeny of *FABP* genes across a wide range of phyla. In this study, we analyzed the number, structure, chromosomal location, and phylogeny of *FABP* genes in 18 organisms from *C.* *elegans* to *Homo* *sapiens*. A total of 12 types of *FABP* genes were identified in the 18 species, and no single organism exhibited all 12 fatty acid‐binding genes (*FABPs*). The absence of a specific *FABP* gene in tissue may be related to the absence of fat storage in the corresponding tissue. The genomic loci of the *FABP* genes were diverse, and their gene structures varied. The results of the phylogenetic analysis and the observation of conserved gene synthesis of FABP family genes/proteins suggest that all *FABP* genes may have evolved from a common ancestor through tandem duplication. This study not only lays a strong theoretical foundation for the study of fat deposition in different organisms, but also provides a new perspective regarding metabolic disease prevention and control and the improvement of agricultural product quality.

## Graphical abstract

To clarify the relationships between fatty acid‐binding proteins (FABPs) and the tissues in which fat is deposited, the number, gene structure, conserved synteny, and evolution of FABP family genes in 18 species were systemically studied. In total, 142 FABPs were identified. The absence of a specific FABP gene in tissue may be related to the absence of fat storage in the corresponding tissue.

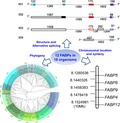

**Abbreviations**
*fabps*: fish and invertebrates’ fatty acid‐binding genes
fabps: fish and invertebrates’ fatty acid‐binding proteins
*FABPs*: mammalian fatty acid‐binding genes
FABPs: mammalian fatty acid‐binding proteins
LBP: lipid‐binding protein
ML: maximum‐likelihood
NJ: neighbor‐joining
WAT: white adipose tissue

From *Caenorhabditis* *elegans* to *Homo* *sapiens*, all animal species have found a way to store excess energy in the form of fat for future needs. Worms (*C.* *elegans*) store fat in the intestine [1, 2], and sharks store fat in the liver [3, 4]. However, in most species, fat is stored mainly in white adipose tissue (WAT) to provide energy during periods when energy demands exceed caloric intake [5]. The location of WAT varies in different species. For example, the most efficient site for WAT is the intra‐abdominal region, as observed in most amphibians and reptiles; in nearly all mammals except for pinnipeds (seals) and certain small cetaceans (whales and dolphins) and in many birds, adipose tissue is partitioned into a dozen or more discrete depots that are widely distributed around the body [6]; fish mainly store fat in the liver, muscle, and mesentery; and in the platypus, fat is stored in the tail [7]. Fat distribution plays an important role in the risk of developing metabolic syndrome. Increased intra‐abdominal/visceral fat promotes a high risk of obesity, diabetes, and other metabolic diseases, whereas increased subcutaneous fat in the thighs and hips is responsible for little or no risk [2]. Notably, adipose tissues present in different parts of poultry and livestock play an important role in product quality [8].

Many studies have shown that the storage and distribution of fat are related to age, sex, hormones, fat synthesis, fat decomposition transport, and other factors [9, 10, 11, 12]. Among these factors, fatty acid‐binding proteins (FABPs) are critical mediators of fat storage and distribution [13, 14, 15, 16] that bind fatty acids and other lipid ligands. Epidermal FABPs (EFABPs) are differentially expressed between human omental and subcutaneous adipose tissue [11], and elevated levels of adipocyte FABPs (AFABPs) have been found in pericardial fat tissue and are associated with cardiac dysfunction in obese people [17]. AFABP and HFABP (muscle and heart FABP) have been considered candidate genes for pig fatness traits. AFABP is involved in the regulation of intramuscular fat accretion in mammals such as Duroc pigs, mice, chickens, and rabbits [18, 19, 20].

Fatty acid‐binding proteins are a family of small cytosolic proteins that bind hydrophobic ligands (mainly fatty acids) noncovalently. They are 14‐ to 15‐kDa proteins of 126–134 amino acids and are named after the first tissue from which they are isolated or identified [12]. There are 12 known FABPs in vertebrate and invertebrate animals. In mammals, nine different FABPs with a tissue‐specific distribution have been identified: FABP1 (LFABP, liver), FABP2 (IFABP, intestinal), FABP3 (HFABP, muscle and heart), FABP4 (AFABP, adipocyte), FABP5 (EFABP, epidermal), FABP6 (IlFABP, ileal), FABP7 (BFABP, brain), FABP8 (MFABP, myelin), and FABP9 (TFABP, testis) [21]. In teleost fish, two members of the FABP family, FABP10 (Lb‐FABP, liver basic FABP) and FABP11, have been discovered [22]. In addition, FABP12 has been identified in humans, rats, and mice [23]. Despite the variable sequence identity of these proteins, whose amino acid sequence similarity ranges from 20% to 70%, all FABPs share a conserved tertiary structure composed of 10 antiparallel beta‐sheets and two alpha‐helixes. The orthogonal β‐sheets wrap around a large solvent‐accessible ligand‐binding cavity centered at one end of the barrel, where the helix–turn–helix motif is proposed to act as a ‘portal’ to allow ligand entry [24].

Evolutionary studies have shown that FABPs evolved via successive gene duplications, generating a large number of tissue‐specific homologs [24, 25, 26]. However, the systematic investigation of *FABP* genes in organisms with a heterogeneous fat distribution in which fat is located in various organs remains in its infancy. The number, structure, sequence identity, and phylogeny of *FABP* genes in these organisms are unclear. Thus, based on the complete genomes of *C.* *elegans*, *Callorhinchus* *milii* [27], and other organisms, we systematically analyzed FABP gene organization in 18 species, including organisms with a heterogeneous fat distribution and important domestic economic species (Table 1)*.* We found that although 12 *FABP* genes have been identified in the selected species, not all types of fatty acid‐binding genes (*FABPs*) are found in certain organisms. In addition to the ‘canonical’ *fabp* gene structure including four exons and three introns, some atypical protein‐coding genes exist, including seven exon–six intron, five exon–four intron, and three exon–two intron genes. Alternative splicing, a common phenomenon in humans and other species, greatly increases the diversity of FABP proteins. Based on the phylogenetic analysis and gene synthesis of FABP family genes/proteins, we verified that all *FABP* genes could have evolved from an ancestral *FABP* gene through gene duplication. In this study, the differences in fat deposition sites between different species were considered in terms of the transport of fatty acids. This work provides a new research hypothesis and direction for the study of ectopic fat deposition in humans and domestic economic species.

**Table 1:** Distribution of FABPs in organisms with a heterogeneous fat distribution. The upper or lower part of the gene nomenclature refers to the Ensembl database. N, could not be identified.

	FABPs^a^												
Organisms	LFABP (FABP1)	IFABP (FABP2)	HFABP (FABP3)	AFAPB (FABP4)	EFABP (FABP5)	ILFABP (FABP6)	BFABP (FABP7)	MFABP (FABP8)	TFABP (FABP9)	Lb‐FABP (FABP10)	FABP11	FABP12	Gene number
Human (*H. sapiens*)	*FABP1*	*FABP2*	*FABP3*	*FABP4*	*FABP5*	*FABP6*	*FABP7*	*FABP8*	*FABP9*	N	N	*FABP12*	10
Rat (*R. norvegicus*)	*FABP1*	*FABP2*	*FABP3*	*FABP4*	*FABP5*	*FABP6*	*FABP7*	*FABP8*	*FABP9*	N	N	*FABP12*	10
Mouse (*M. musculus*)	*FABP1*	*FABP2*	*FABP3*	*FABP4*	*FABP5*	*FABP6*	*FABP7*	*FABP8*	*FABP9*	N	N	*FABP12*	10
Cow (*B. taurus*)	*FABP1*	*FABP2*	*FABP3*	*FABP4*	*FABP5*	*FABP6*	*FABP7*	*FABP8*	*FABP9*	N	N	*FABP12*	10
Pig (*S. scrofa*)	*FABP1*	*FABP2*	*FABP3*	*FABP4*	*FABP5*	*FABP6*	*FABP7*	*FABP8*	*FABP9*	N	N	N	9
Dolphin (*T. truncatus*)	*FABP1*	*FABP2*	*FABP3*	*FABP4*	*FABP5*	*FABP6*	N	*FABP8*	N	N	N	N	7
Platypus (*O. anatinus*)	*FABP1*	*FABP2*	N	N	*FABP5*	N	N	*FABP8*	N	N	N	N	4
Chicken (*G. gallus*)	*FABP1*	*FABP2*	*FABP3*	*FABP4*	*FABP5*	*FABP6*	*FABP7*	*FABP8*	N	*FABP10*	N	N	9
Xenopus (*X. tropicalis*)	*fabp1*	*fabp2*	*fabp3*	*fabp4*	N	*fabp6*	*fabp7*	*fabp8*	N	*fabp10*	N	N	8
Anole lizard (*A. carolinensis*)	*FABP1*	*FABP2*	N	*FABP4*	*FABP5*	*FABP6*	*FABP7*	*FABP8*	N	*FABP10*	N	N	8
Zebrafish (*D. rerio*)	*fabp1a*, *fabp1b.1*, *fabp1b.2*	*fabp2*	*fabp3*	N	N	*fabp6*	*fabp7a* *fabp7b*	N	N	*fabp10a, fabp10b*	*fabp11a*, *fabp11b*	N	12
Tilapia (*O. niloticus*)	*fabp1b.1*	*fabp2*	*fabp3*	N	N	*fabp6*	*fabp7a*	N	N	*fabp10a*, *fabp10b*	*fabp11a*, *fabp11b*	N	9
Fugu (*T. rubripes*)	*fabp1b.1*	*fabp2*	*fabp3*	N	N	*fabp6*	*fabp7* *fabp7a*	N	N	*fabp10a* *fabp10b*	*fabp11a*	N	9
Medaka (*O. latipes*)	*fabp1b.1*	*fabp2*	*fabp3*	N	N	*fabp6*	*fabp7*, *fabp7a*	N	N	*fabp10a*, *fabp10b*	*fabp11a*, *fabp11b*	N	10
Shark (*C. milii*)	ENSCMIG00000013098, ENSCMIG00000013107	ENSCMIG00000003354	ENSCMIG00000017535	N	N	N	ENSCMIG00000011741	N	N	ENSCMIG00000016027	N	N	6
Ciona *(C. intestinalis)*	ENSCING00000012923	ENSCING00000014756	N	N	N	ENSCING00000014865	ENSCING00000002061	ENSCING00000005575	N	N	N	N	5
Fruit fly (*D. melanogaster*)	*Fabp*												1
Worm (*C. elegans*)	*lbp‐5*, *lbp‐6*, *lbp‐7*, *lbp‐8*, *lbp‐9*												5

^a^ The upper or lower part of gene nomenclature refers to Ensembl database.

## Results and Discussion

### *FABP* family gene identification from *C.* *elegans* to *Homo* *sapiens*

Fatty acid‐binding proteins are members of the superfamily of lipid‐binding proteins (LBPs). The primary role of all FABP family members is the regulation of fatty acid uptake and intracellular transport. Thus, we wondered how many *fabp* genes exist in species with different adipose tissue depots to regulate fat transport and storage. We found that the presence or absence of 12 *FABP* genes (*FABP1‐FABP12*) varies from *C.* *elegans* to *H.* *sapiens* (Table 1). Among these genes, *FABP1* and *FABP2* are conserved in 16 chordates, from *Ciona* *intestinalis* to *H.* *sapiens*. *FABP3*, *FABP6*, and *FABP7* are conserved in vertebrate genomes. *FABP4*, *FABP5*, and *FABP8* have been identified in humans, mice, birds, amphibians, and reptiles but not in fish. *FABP9* and *FABP12* are found only in mammals. In contrast to *FABP10* in chickens, amphibians, reptiles, and fish, *FABP11* has only been identified in teleosts. On the other hand, some species exhibit limited copies of *FABPs* such as *fbpb1*, *fabp2*, *fabp5*, *fabp6*, *fabp7*, and *fabp8*. For example, four *FABPs* (*FABP1*, *FABP2*, *FABP5*, and *FABP8*) are found in the genome of the platypus (*Ornithorhynchus* *anatinus*). According to Ensembl, live‐like, intestinal‐type, brain‐like, and myelin P2 protein‐like fish and invertebrates’ fatty acid‐binding genes (*fabps*) exist in *C.* *intestinalis*. In *Drosophila* *melanogaster,* only one *fabp* gene can be found*.* In *C.* *elegans*, there are nine *lbp* genes, *lbp‐1* to *lbp‐9*. *lbp‐1*, *lbp‐2*, *lbp‐3*, and *lbp‐4* are exclusive to *C.* *elegans*, and their products localize to the extracellular region. These four genes are not orthologs of *FABP1*, *FABP2*, *FABP3*, and *FABP4* in mammals. Based on the HomoloGene annotation of the NCBI (https://www.ncbi.nlm.nih.gov/homologene), *lbp* *‐5*,* lbp‐6*,* lbp‐7*, and *lbp‐*8 are orthologs of *FABP4* in mammals. *lbp‐9* is an ortholog of FABP8 in humans and *FABP11a/11b* in fish. With the updated shark (*C. milii*) genome, we identified five *fabps* (*fabp1*,* fabp2*,* fabp3*,* fabp7*, and *fabp10*) in cartilaginous fish. *fabp1*,* fabp2*,* fabp3*,* fabp6*,* fabp7*,* fabp10*, and *fabp11* have been identified in the genomes of teleost fish. More gene copies have appeared in teleost fish, followed by divergence. For example, in zebrafish, there is only one copy of *fabp2*,* fabp3*, and *fabp6*, while there are three copies of the *fabp1* gene (*fabp1a*,* fabp1b.1*, and *fabp1b.2*), and there are two copies of *fabp7*, *fabp10,* and *fabp11*. In total, 142 *FABPs* were identified in the 18 collected organisms (Tables 1 and S1). In Table S1, we list the gene annotations, including the gene ID, transcript ID, splice variant number, RNA/protein length, exon/intron number, and differing genome sites.

Considering the relationship between FABP genes and fat deposit tissue, we propose that the absence of the *FABP5* (EFABP, epidermal) gene in Xenopus and fish may be related to the absence of subcutaneous WAT in these species. In addition, the lack of the *FABP4* (AFABP, adipocyte) gene in fish may account for the deposition of fat in the liver, muscle, and mesentery in most fish rather than in adipocytes. Indeed, in zebrafish, it has been proven that *fabp11a*, and not *fabp4*, plays a key role during adipogenesis [28, 29]. However, the mechanism regulating the differential distribution of fat is complex, and for many FABPs, the precise physiological function is not completely understood; thus, how FABPs regulate the fat deposited in different tissues also requires further study.

### Diversity of the gene structure of FABPs

**Fig. 1:**
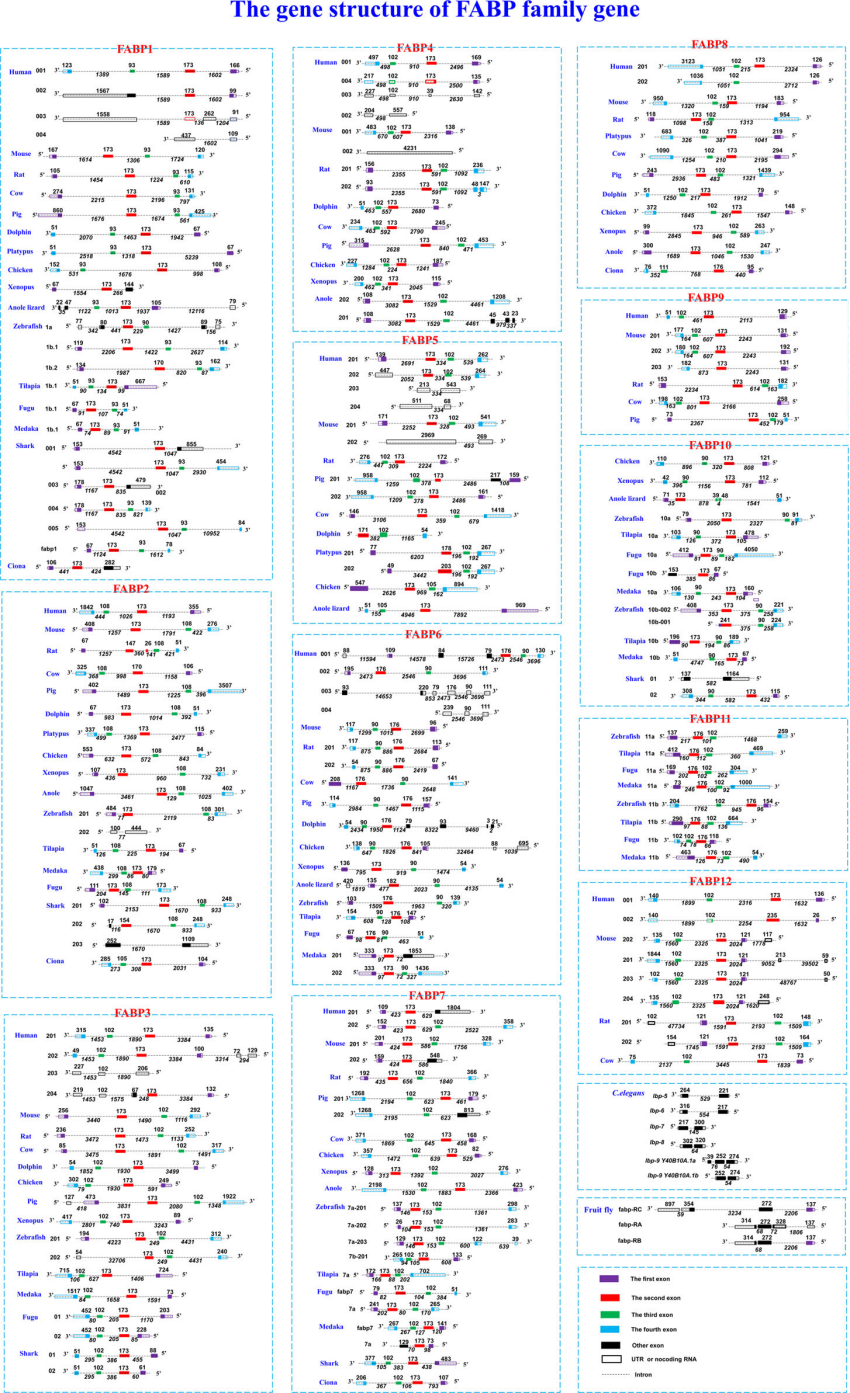
The gene structures of 12 *FABPs* in 18 organisms. Transcripts are drawn as boxes (exons) and lines connecting the boxes (introns). Filled boxes represent coding sequences, and unfilled boxes (or portions of boxes) represent UTRs. The length of the exons is provided on the boxes, and the length of the introns is provided under the corresponding lines. This view shows all spliced transcripts for a gene, including EST transcripts and ncRNAs (noncoding RNAs).

As reported previously, most functional *FABP* genes exhibit a ‘canonical’ structure including four exons and three introns [26]. However, some protein‐coding genes with an atypical structure also exist (Figs 1, S1 and Table 2). For example, seven exons and six introns are found in *FABP6* of dolphin (*Tursiops truncates*); there are five exons and four introns in *FABP2* of *Rattus norvegicus* and *FABP10* of *Anolis carolinensis,* respectively; there are three exons and two introns in *FABP5* of *T. truncates*, *fabp1* of *Xenopus tropicalis*, *fabp10b* of *Takifugu rubripes*, *fabp7a *of* Oryzias latipes*, and *FABP1* of *C. intestinalis*; and in *C. elegans*,* lbp‐5*,* lbp‐6*,* lbp‐7*, and *lbp‐8* are composed of two exons and one intron. In addition, alternative splicing may result in two (*FABP6* and *FABP7* of humans), two (*FABP5* and *FABP7* of pigs), one (*fabp6* of medaka), three (*fabp1*,* fabp2*, and *fabp10* of sharks), or one (*lbp‐9* of *C. elegans*) protein encoded by genes exhibiting an unrepresentative gene structure (Fig. 1 and Table 2). Additionally, some lncRNAs, retained introns, and nonsense‐mediated decay are observed in *FABP* genes derived from alternative splicing (Figs 1, S1, and Table S1).

**Table 2:** List of protein‐coding genes with an unrepresentative gene structure.

Organism‐types	Ensembl gene ID	Location	Splice variants	Transcript ID	bp	Protein	Exons	Introns
Rat‐FABP2	ENSRNOG00000024947	2:227,080,924‐227,083,501:1	1	ENSRNOT00000029871.3	399	132aa	5	4
Dolphin‐FABP5	ENSTTRG00000005377	scaffold_98333:298,656‐300,529:1	1	ENSTTRT00000005366.1	327	108aa	3	2
Dolphin‐FABP6	ENSTTRG00000012640	scaffold_106877: 69,634‐93,441:‐1	1	ENSTTRT00000012640.1	516	171aa	7	6
Xenopus‐fabp1	ENSXETG00000001938	GL172916.1: 603,458‐605,661:1	1	ENSXETT00000004132.3	384	128aa	3	2
Anole‐FABP10	ENSACAG00000005346	GL343498.1: 40,727‐43,888:1	1	ENSACAT00000005335.3	382	125aa	5	4
Fugu‐ fabp10b	ENSTRUG00000020508	12:585,852‐586,715:‐1	1	ENSTRUT00000051675.1	393	130aa	3	2
Medaka‐fabp7a	ENSORLG00000013475	22:8,108,362‐8,108,904:‐1	1	ENSORLT00000016902.2	375	132aa	3	2
Ciona ‐fabp1	ENSCING00000014865	7: 5,911,360‐5,912,785:1	1	ENSCINT00000026940.2	561	128aa	3	2
*C. elegans*‐lbp5	WBGene00002257	I: 6,731,269‐6,732,280:‐1	1	W02D3.7	485	136aa	2	1
*C. elegans*‐lbp6	WBGene00002258	I: 6,728,290‐6,729,376:‐1	1	W02D3.5	533	135aa	2	1
*C. elegans*‐lbp7	WBGene00002259	V: 13,879,552‐13,880,213 :1	1	T22G5.2	517	137aa	2	1
*C. elegans*‐lbp8	WBGene00002260	V: 13,890,679‐13,891,364 :1	1	T22G5.6	622	137aa	2	1
Human‐FABP6	ENSG00000170231	5:160,187,367‐160,238,735:1	4	ENST00000393980.8	756	177aa	7	6
Human‐FABP7	ENSG00000164434	6:122,779,716‐122,784,074:1	2	ENST00000356535.4	2086	166aa	3	2
Pig‐FABP5	ENSSSCG00000006153	4:55,276,621‐55,282,908:‐1	2	ENSSSCT00000054753.1	1740	135aa	5	4
Pig ‐FABP7	ENSSSCG00000004234	1:39,871,298‐39,876,298:‐1	2	ENSSSCT00000038263.1	2183	132aa	3	2
Medaka‐fabp6	ENSORLG00000012622	17:19,211,982‐19,214,509:1	2	ENSORLT00000047118.1	2359	164aa	3	2
Shark‐fabp1	ENSCMIG00000013098	KI635913.1:4,310,897‐4,329,941:1	5	ENSCMIT00000030904.1	1181	142aa	3	2
Shark‐fabp2	ENSCMIG00000003354	I635873.1:8,109,897‐8,115,283:1	3	ENSCMIT00000006086.1	1361	147aa	2	1
shark‐fabp10	ENSCMIG00000016027	KI635890.1:6,798‐9,285:‐1	2	ENSCMIT00000038686.1	1301	135aa	2	1
*C. elegans*‐lbp9	WBGene00021486	V: 2,068,449‐2,069,143:1	2	Y40B10A.1a	565	152aa	3	2

As shown in Fig. 1, which shows the gene structure of 142 *fabps*, we found that the second and third exons are conserved in almost all protein‐coding *FABP* genes with four exons and three introns. Although the exon/intron positions are similar in all genes, the length, especially that of the intron, is variable. For example, the lengths of the first intron of *FABP1* vary from 74 bp (*FABP1* in medaka) to 5239 bp (*FABP1* in platypus) because of the differences in genome size among different species. In contrast, the length of exons varies relatively little. In almost all *FABPs*, the length of the second exon is 173 bp, except in *FABP11*, in which the length of the second exon is 176 bp. The third exon of most *FABPs *(*FABP3*,* FABP4*,* FABP5*,* FABP7*,* FABP8*,* FABP9*,* FABP11*, and *FABP12*) consists of 102 bp nucleotides; however, there are 93, 108, 90, and 90 bp nucleotides in the third exons of *FABP1*,* FABP2*,* FABP6*, and *FABP10*, respectively (Fig. 1).

### Alternative splicing of *FABP* genes

Alternative splicing is a common phenomenon in eukaryotes that greatly increases the diversity of proteins that can be encoded by genes [30]. For example, in humans, ~ 95% of multiexon genes undergo alternative splicing [31]. According to the Ensembl annotation, 33 of the 142 *FABPs* (Fig. 1 and Table S2) exhibit splice variants, except those in the genomes of *Bos taurus*, *T. truncates*,* Gallus gallus*,* X. tropicalis*,* Oreochromis niloticus*, and *C. intestinalis*. Notably, in humans, with the exception of *FABP2* and *FABP9,* eight other *FABP* genes exhibit alternative splicing; *FABP1* and *FABP3* to *FABP6* exhibit four splice variants, whereas *FABP7*,* FABP8*, and *FABP12* exhibit two splice variants. In addition, in *D. melanogaster*, there is only one *FABP* gene, but it exhibits three splice variants, with all transcripts retaining the second exon during alternative splicing (Fig. 1). Mapping these variants to those in mammals, we found that the second and third exons in mammals are combined to form the second exon (272 bp) in *D. melanogaster*. Therefore, exon skipping and intron retention are involved in *fabp* post‐transcriptional splicing.

### The conserved synteny of *FABP* genes

**Fig. 2:**
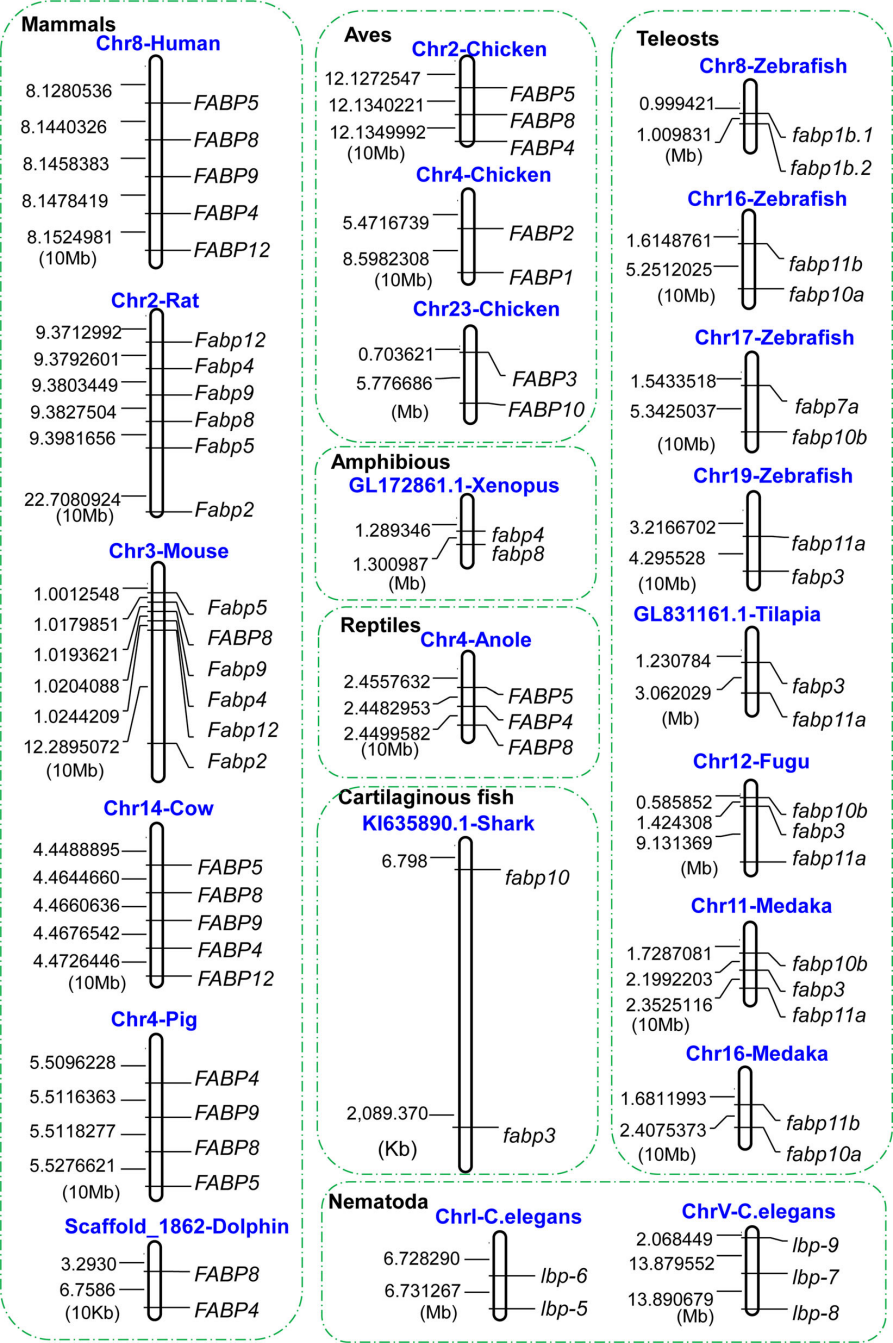
*FABP* linkage map on chromosomes or scaffolds. The numbers on the left are the *FABP* starting points, which were derived from the genome sequences in the Ensembl Genomes database.

Conserved synteny (the colocalization of genes on chromosomes) is sometimes used to describe the preservation of the precise order of genes on a chromosome passed down from a common ancestor [32], although many geneticists reject this use of the term [33]. Many studies have indicated that all FABPs are likely to have arisen from common ancestral genes through duplication and diversification [25, 34, 35]. Thus, we analyzed the synteny of *FABP* family genes. As shown by the genetic linkage maps in Fig. 2, we found that *FABP4* and *FABP8* are always located on the same chromosome in mammals, aves, and amphibians. In mammals, *FABP4*,* FABP5*,* FABP8*,* FABP9*, and *FABP12* are arranged in sequential order on human chromosome 8, rat chromosome 2, mouse chromosome 3, and cow chromosome 14, respectively. In mice, *FABP2* is also located on chromosome 3, slightly separated from *FABP4*, *FABP5*, *FABP8*, *FABP9,* and *FABP12*. In chickens, *FABP4*, *FABP5,* and *FABP8* are adjacent to each other on chromosome 2, while *FABP2/FABP1* and *FABP3/FABP10* are paired on chromosomes 4 and 23, respectively. The sequence arrangement of *FABP4/FABP5/FABP8/FABP9* on a single chromosome and the variation of the *FABP* gene cluster in different tetrapod lineages indicate that the *FABP* family genes may have arisen through a series of unequal crossover events during meiosis, resulting in continuous tandem duplications in vertebrate lineages [36]. In fish, *fabp3* is clustered with *fabp10* or/and *fabp11*. For example, in sharks, *fabp3/fabp10* are colocalized on scaffold KI635890.1, but in zebrafish and tilapia, *fabp3/fabp11a* are located on chromosome 19 and scaffold GL81161.1, respectively; however, in takifugu and medaka, *fabp3/fabp10b/11a* are located on chromosomes 12 and 11, respectively. In zebrafish, the duplicated *fabp1* genes *fabp1b.1* and fabp1b.2 form a gene cluster on chromosome 8. In *C. elegans*, the *lbp5/lbp6* and *lbp7/lbp8/lbp9* gene clusters are positioned on chromosomes I and V (Fig. 2), respectively, suggesting that they might have arisen via tandem gene duplication.

### Phylogenetic relationship between the FABPs of organisms with a heterogeneous fat distribution

Thus far, FABP genes have only been found in vertebrates and invertebrates, and evolutionary studies have distinguished major subfamilies that could have been derived from a single ancestral gene close to the time of the vertebrate/invertebrate split [12]. In this work, to understand the evolutionary relationships of organisms with a heterogeneous fat distribution, we downloaded all selected genes encoding proteins, which varied in length from 108 to 189 amino acids. Multiple sequence alignment of proteins using ClustalW showed that the identities of the proteins ranged from 8.8% (fabp1 in shark vs. fabp5 in dolphin) to 96.9% (FABP7 in mouse vs. FABP7 in rat). The similarity of the amino acid sequences of FABPs of the same type in different organisms is greater than that between FABPs of different types in the same organism. For example, FABP1 from 18 different species consistently displays sequence identities higher than 60%, while the identities of 10 FABPs (FABP1‐FABP9, FABP10) in the human genome are as low as 23% on average (Table S3).

**Fig. 3:**
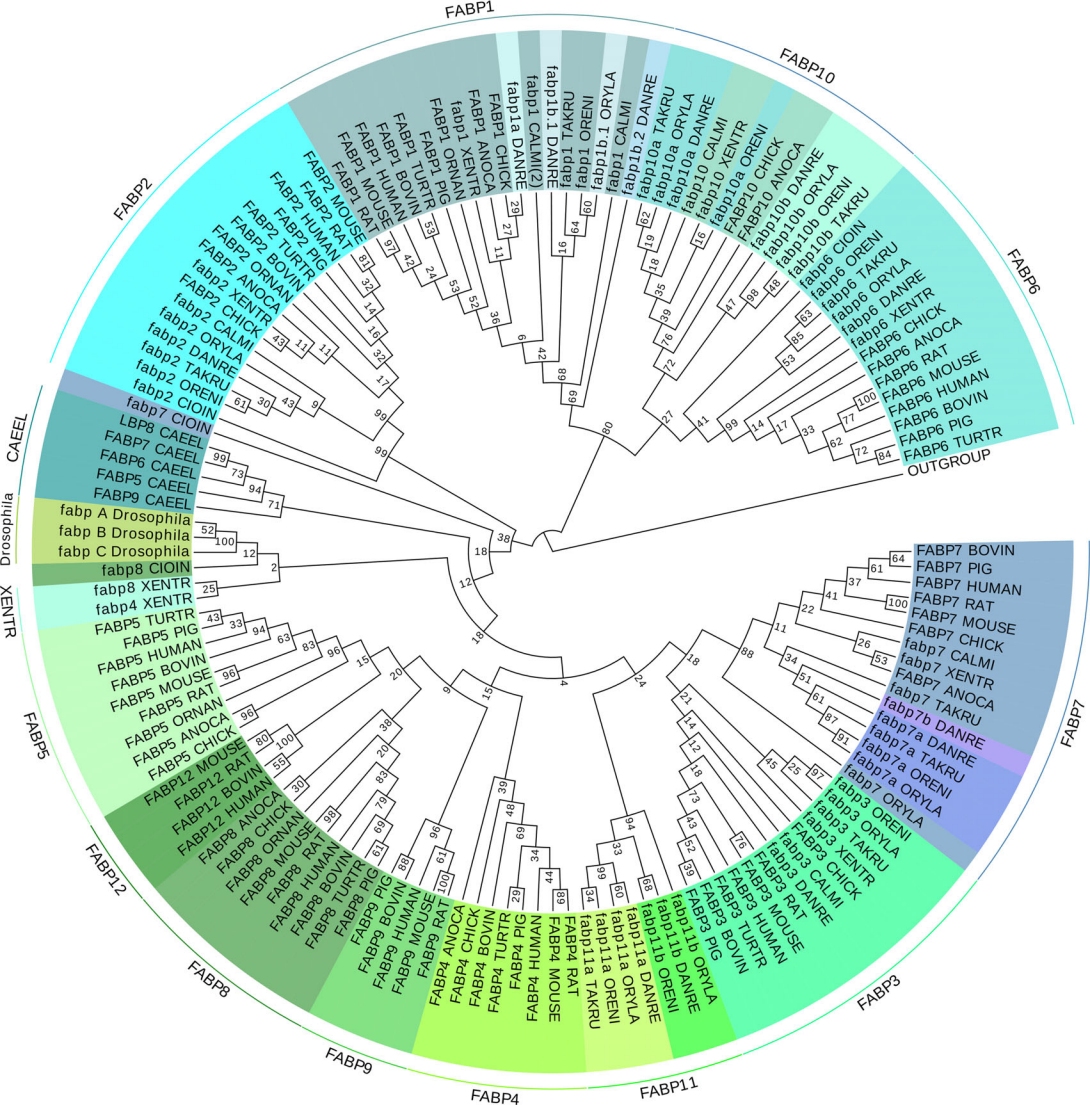
Phylogenetic tree showing the evolution of FABP family proteins. The amino acid sequences of FABP proteins were used for phylogenetic analysis based on the NJ method, and relationships were assessed using bootstrap values (×1000). The amino acid sequences used in this tree include FABP1 (P07148), FABP2 (P12104), FABP3 (P05413), FABP4 (P15090), FABP5 (Q01469), FABP6 (P51161), FABP7 (O15540), FABP8 (P02689), FABP9 (Q0Z7S8), and FABP12 (A6NFH5) in human; FABP1 (P02692), FABP2 (A0A0A0MY01), FABP3 (P07483), FABP4 (Q5XFV4), FABP5 (P55053), FABP6 (G3V6H6), FABP7 (P55051), FABP8 (D3ZFG5), FABP9 (P55054), and FABP12 (B7SUM8) in rat; FABP1 (P12710), FABP2 (P55050), FABP3 (P11404), FABP4 (P04117), FABP5 (Q05816), FABP6 (P51162), FABP7 (P51880), FABP8 (P24526), FABP9 (O08716), and FABP12 (Q9DAK4) in mice; FABP1 (P80425), FABP2 (F1MFF7), FABP3 (P10790), FABP4 (F1MHQ4), FABP5 (P55052), FABP6 (A0A452DID8), FABP7 (Q09139), FABP8 (P02690), FABP9 (F1MRS8), and FABP12 (G3N125) in cow (*B. taurus,* BOVIN); FABP1 (A0A2C9F382), FABP2 (Q45KW7), FABP3 (O02772), FABP4 (O97788), FABP5 (Q2EN74), FABP6 (F1RR40), FABP7 (A0A287ABK1), FABP8 (P86412), and FABP9 (A0A287ALB1) in pigs; FABP1 (ENSTTRT00000006950.1), FABP2 (A0A2U3V5V8), FABP3 (ENSTTRT00000014872.1), FABP4 (ENSTTRT00000006961.1), FABP5 (ENSTTRT00000005366.1), FABP6 (ENSTTRT00000012640.1), and FABP8 (ENSTTRT00000006950.1) in Dolphin (*T. truncatus*, TURTR); FABP1 (F7B3U2), FABP2 (F7AEJ8), FABP5 (F7FRZ0), and FABP8 (F7GA78) in platypuses (*O. anatinus,* ORNAN); FABP1 (Q90WA9), FABP2 (Q7ZZZ5), FABP3 (F1NUQ3), FABP4 (A0A1D5NYF3), FABP5 (A0A1D5PPQ2), FABP6 (F1NUJ7), FABP7 (Q05423), FABP8 (A0A1D5PJZ5), and FABP10 (A0A140T8G0) in chickens; fabp1 (F6SCM6), fabp2 (F6YX57), fabp3 (F6W709), fabp4 (F6YY49), fabp6 (F6SPQ9), fabp7 (Q28CE2), fabp8 (F6YY42), and fabp10 (F6QM14) in Xenopus (*X. tropicalis*, XENTR); FABP1 (G1KHU4), FABP2 (G1KNM3), FABP4 (R4GB14), FABP5 (G1KNY2), FABP6 (H9G789), FABP7 (G1K9I3), FABP8 (G1KNY3), and FABP10 (H9GA34) in anole lizard (*A. carolinensis,* ANOCA); fabp1a (Q1AMT3), fabp1b.1 (Q4VBT1), fabp1b.2 (A0A0R4IRM6), fabp2 (Q9PRH9), fabp3 (Q8UVG7), fabp6 (Q6IMW5), fabp7a (Q9I8N9), fabp7b (Q6U1J7), fabp10a (Q9I8L5), fabp10b (X1WFK9), fabp11a (Q66I80), and fabp11b (Q503X5) in zebrafish (*D. rerio*, DANRE); fabp1b.1 (I3KDH3), fabp2 (I3KM58), fabp3 (I3J1Z5), fabp6 (I3JR85), fabp7a (I3IYI6), fabp10a (I3KHE7), fabp10b (I3K8I9), fabp11a (I3J359), and fabp11b (I3J4S9)in tilapia (*O. niloticus*, ORENI); fabp1b.1 (H2USJ2), fabp2 (H2UHN0), fabp3 (A0A3B5K3E7), fabp6 (H2SDA8), fabp7 (H2TV98), fabp7a (A0A3B5K4R2), fabp10a (A0A3B5K3Y6), fabp10b (A0A3B5KPV3), and fabp11a (H2UKI9) in fugu (*T. rubripes*, TAKRU); fabp1b.1 (H2LFL3), fabp2 (H2LGU3), fabp3 (H2M7N9), fabp6 (H2MB86), fabp7 (Q2PHF0), fabp7a (H2ME97), fabp10a (H2MIR1), fabp10b (H2LU71), fabp11a (A0A3B3HSE3), and fabp11b (H2LWA3) in medaka (*O. latipes*, ORYLA); fabp1 (K4G6D8), fabp1 (V9LJ13), fabp2 (K4G5P6), fabp3 (ENSCMIP00000042192), fabp7 (ENSCMIP00000026917), and fabp10 (K4GCB5) in sharks (*C. milii*, CALMI); fabp2 (F6W8U8), fabp6 (F7BFW8), fabp7 (F6SU68), and fabp8 (F6U4M6) in Ciona (*C. intestinalis,* CIOIN); fabpB (NP_001027180.1), fabpC (NP_001027179.1), and fabpA (NP_001027181.1) in fruit fly (*D. melanogaster*).

We further constructed a protein neighbor‐joining (NJ) tree using mega6 (Biodesign Institute, Tempe, AZ, USA ) [37]. As shown in the NJ tree (Fig. 3), all FABPs were split into two clades: FABP1, FABP6, and FABP10 cluster in one clade, and FABP2, FABP3, FABP4, FABP5, FABP7, FABP8, FABP9, FABP11, and FABP12 colocalize in another clade. Moreover, the invertebrate FABPs of Nematoda and Drosophila cluster into the FABP2/FABP3/FABP4/FABP5/FABP7/FABP8/FABP9/FABP11/FABP12 clades, which suggests that the FABP1/FABP6/FABP10 gene cluster may have diverged from another cluster before vertebrate/invertebrate divergence. In teleost fish, fabp7a/fabp7b and fabp11a/fabp11b duplicates share a common node, but fabp1a/fabp1b and fabp10a/fabp10b do not share a common node, although all of these sequences cluster in the same clade with all other sister fish and invertebrates’ fatty acid‐binding proteins (fabps). However, fabp7 and fabp8 in *C. intestinalis* do not cluster into the corresponding clade with other species. The topology of the maximum‐likelihood (ML) tree (Fig. S2) is similar to that of the NJ tree. Together with the conserved *FABP* gene synteny analysis, we confirmed that the current FABP family gene/protein set might have resulted from multiple rounds of duplications and splicing editing divergence during evolution. These results regarding FABP evolution are consistent with the reports of Schaap [25] and M. Wright [35].

As shown by the studies of M. Wright and his colleagues [35, 36, 38, 39, 40, 41], teleost fishes possess many copies of *fabps* genes owing to a whole‐genome duplication even that occurred early in the teleost radiation. In addition, these authors proposed that the two copies of *fabp7a/fabp7b*, *fabp10a/fabp10b,* and *fabp11a/fabp11b* in the teleost fish genome may have resulted from a fish‐specific duplication event [29, 39], although the duplicated zebrafish *fabp1b.1* and *fabp1b.2* sequences are tandemly arrayed on chromosome 8. The *fabp1b.1* and *fabp1b.2* genes of zebrafish are paralogs that were presumably duplicated by unequal crossing‐over during meiosis [35].

## Conclusions

To clarify the relationships between FABPs and the tissues in which fat is deposited, the number, gene structure, conserved synteny, and evolution of FABP family genes in 18 species were systemically studied. There are ten, nine, eight, eight, seven, five, five, one, and five types of FABP family genes in mammals, chickens, Xenopus, anole lizards, teleosts, sharks, Ciona, fruit flies, and worms, respectively (Table 1). In total, 142 FABPs were identified in the selected species (Tables 1 and Table S1). We propose that the loss of a particular FABP may be related to the lack of fat storage in the corresponding tissue. However, since there are many members of the FABP family, how they individually or interactively regulate the distribution of fat in different tissues requires further study.

## Materials and methods

### FABP orthologs identified from *C. elegans* to *Homo sapiens*

In this study, we chose humans, rats, mice, dolphins, platypuses, chickens, Xenopus, anole lizards, teleost fish, sharks, fruit flies, and worms as representative organisms with a heterogeneous fat distribution, and we chose cows, pigs, tilapia, fugu, and medaka as representatives of domestic economic species. Different FABPs are typically identified based on the reciprocal best hits (RBHs) in two genomes using Ensembl BioMart (http://asia.ensembl.org/biomart/martview/1aaa80b6fabd0febf1bc9c3d3c2fb519). Using human *FABP1*, *FABP2*, *FABP3*, *FABP4*, *FABP5*, *FABP6*, *FABP7*, *FABP8*, *FABP9,* and *FABP12*, we searched for orthologs in the rat (*R. norvegicus*), mouse (*Mus musculus*), cow (*B. taurus*), pig (*Sus scrofa*), dolphin (*Tursiops truncatus*), platypus (*O. anatinus*), chicken (*G. gallus*), Xenopus (*X. tropicalis*), anole lizard (*A. carolinensis*), zebrafish (*Danio rerio*), tilapia (*O. niloticus*), fugu (*T. rubripes*), medaka (*O. latipes*), Ciona (*C. intestinalis*), worm (*C. elegans*), and fruit fly (*D. melanogaster*) genomes with Ensembl BioMart. Similarly, using zebrafish *fabp10a*, *fabp10b*, *fabp11a,* and *fabp11b*, we performed searches for orthologs in the genomes of the 17 other organisms.

As previously reported, FABPs evolved through successive gene duplications, generating a large number of tissue‐specific homologs. However, a high degree of gene duplication, particularly in distantly related organisms, hinders ortholog identification by different methods. To avoid obtaining false orthology from Ensembl Compara, we performed NCBI BLASTP and TBLASTN searches using the default parameters to detect orthology. Then, we manually verified the missing genes using OrthoDB (https://www.orthodb.org/), ZFIN (Zebrafish International Resource Center database, http://zfin.org/), and WormBase (https://wormbase.org/#01-23-6).

### Gene structure and alternative splicing analysis

Gene structure and splicing variant information was obtained by referencing the Ensembl database (http://asia.ensembl.org/index.html). We manually searched and downloaded information about genetic structure and splice variants for each FABP. Then, we drew the gene structures including every *fabps* transcript with PowerPoint (PPT).

### Gene and protein sequence retrieval

Most FABP protein sequences were retrieved from the UniProt database (https://www.uniprot.org/uploadlists/); the other transcript sequences from *T. truncatus*, *C. milii*, *D. melanogaster*, and *C. elegans* were downloaded from Ensembl (http://asia.ensembl.org/index.html) and NCBI (https://www.ncbi.nlm.nih.gov/gene/). The protein lengths of all FABPs ranged from 108 to 189 amino acids.

### FABP genetic linkage map

The chromosomal or scaffold locations of the *FABP* genes were derived from Ensembl genome databases. Based on the provided genetic linkage data, we drew genetic linkage maps using MapDraw 2.1 [42] in Excel.

### Sequence alignments and reconstruction of gene/protein trees

The FABP protein sequences were aligned using the ClustalW algorithm with default parameters and then manually checked. In addition to all the FABP amino acid sequences identified in this study, 144 FABP sequences were also included for phylogenetic analysis. LCN1 (accession number NP_002288), which belongs to the lipocalin family of calycins and has a size (158 amino acids) comparable with those of iLBPs, was used as an outgroup. Phylogenetic trees were constructed using both the NJ and ML methods implemented in mega6 software (bootstrap = 1000) [37]. Evolview v3 [43] was used to visualize and annotate the NJ tree.

## Conflict of interest

All authors declare no conflict of interest.

## Author contributions

YZ conceived and designed the experiments. YZ and JZ performed the experiments. YZ and JZ analyzed the data. YZ, JZ, YR, RL, LY, and GN contributed materials/analysis tools. YZ wrote the paper.

## Supporting information


**Fig. S1.** High‐resolution gene structure in PDF format.

**Fig. S2.** ML tree of FABP proteins.

**Table S1.** 142 FABP gene locations and transcripts. In the table, we list the gene ID, the location on chromosome or scaffold, splice variant numbers, transcript names, transcript IDs, gene length, protein length, biotype and the numbers of exons/introns.

**Table S2.** List of FABP genes subjected to alternative splicing.

**Table S3.** Sequence identity matrix of FABP proteins.
