# Supplementary figures and images for: Tracing the evolution of fatty acid‐binding proteins (FABPs) in organisms with a heterogeneous fat distribution
Source: FEBS Open Bio. 2020 Mar 31;10(5):861–72. doi: 10.1002/2211-5463.12840 (PMC7193176; doi:10.1002/2211-5463.12840)

The gene structure of FABPs gene family

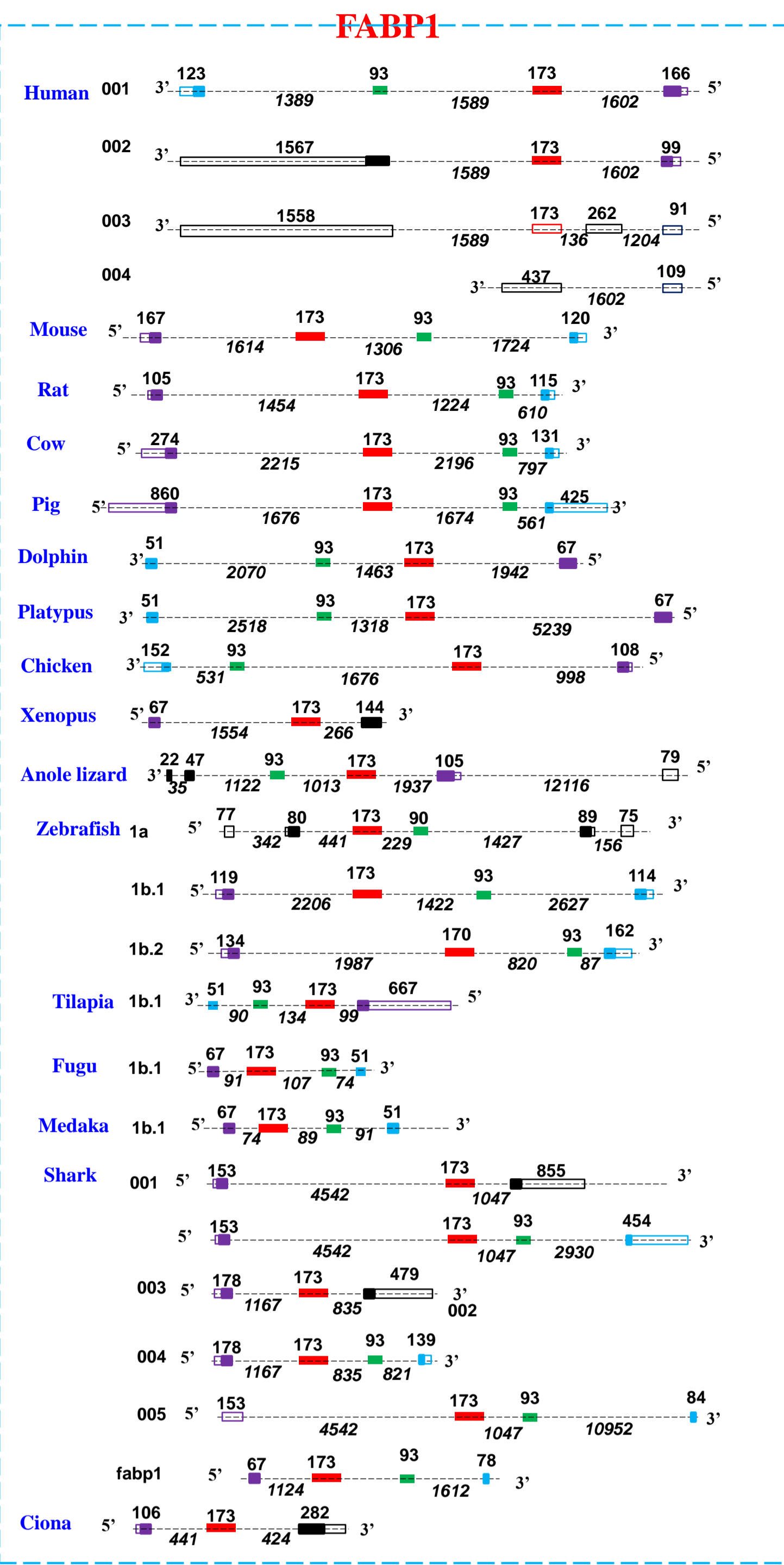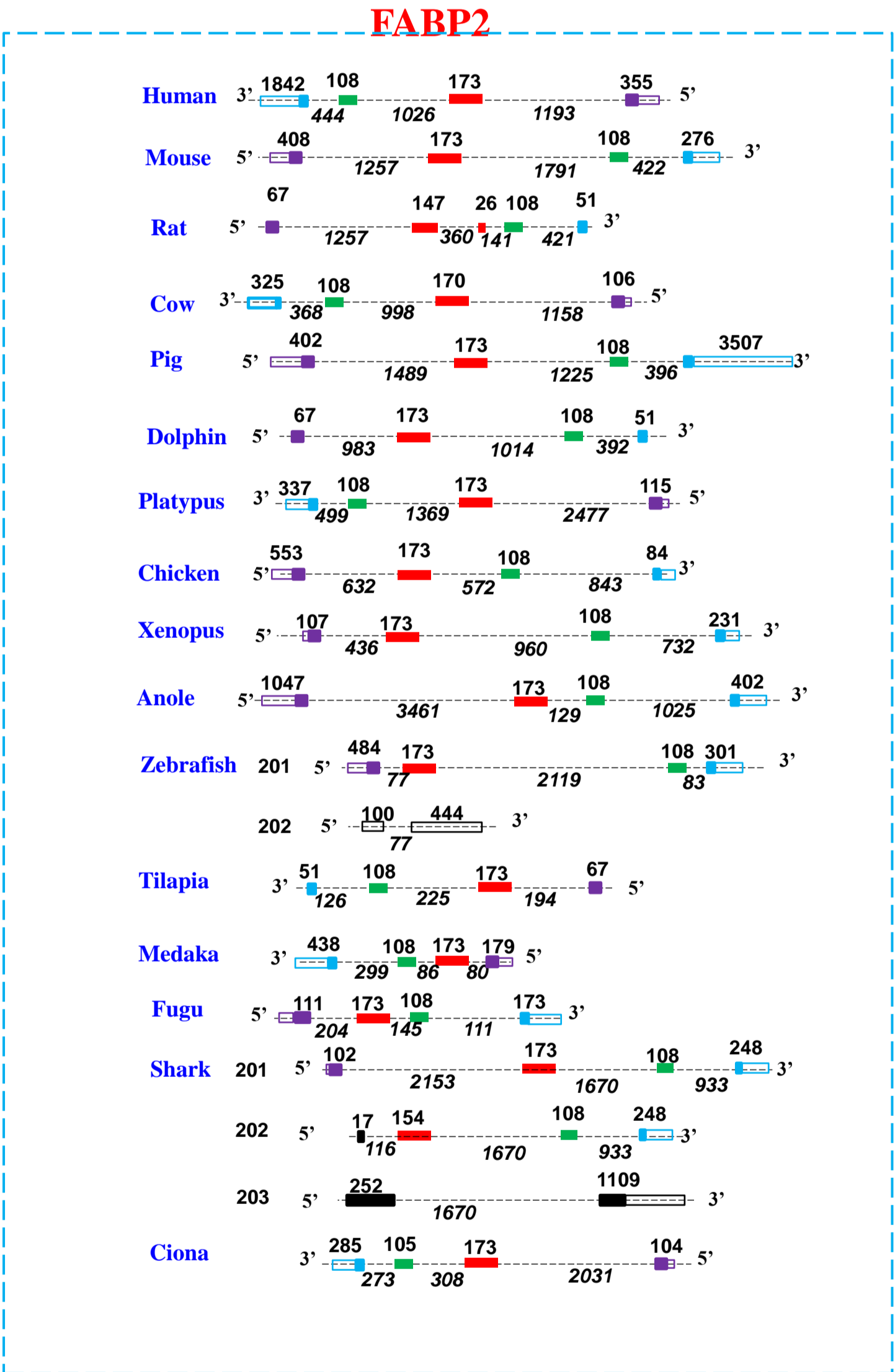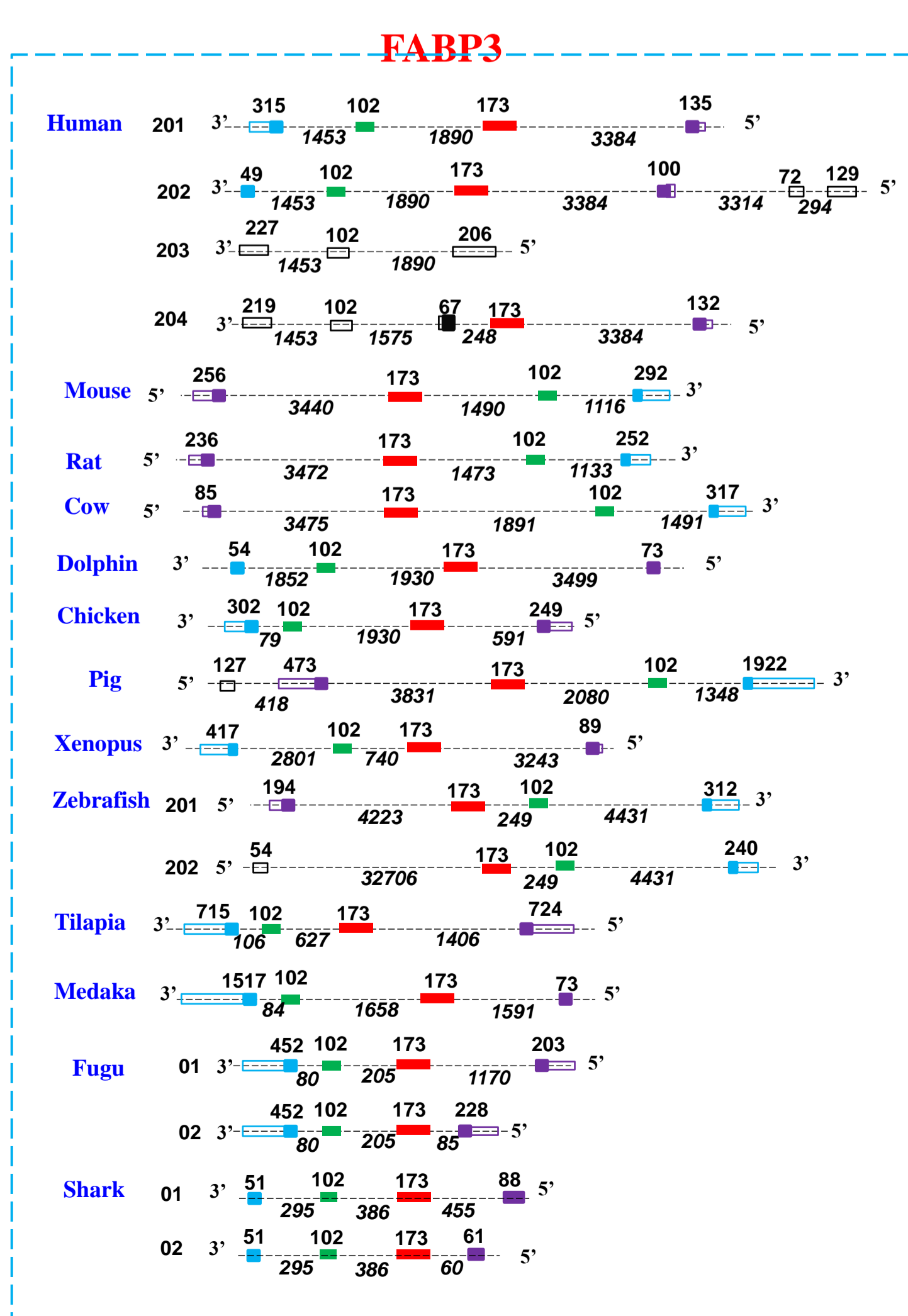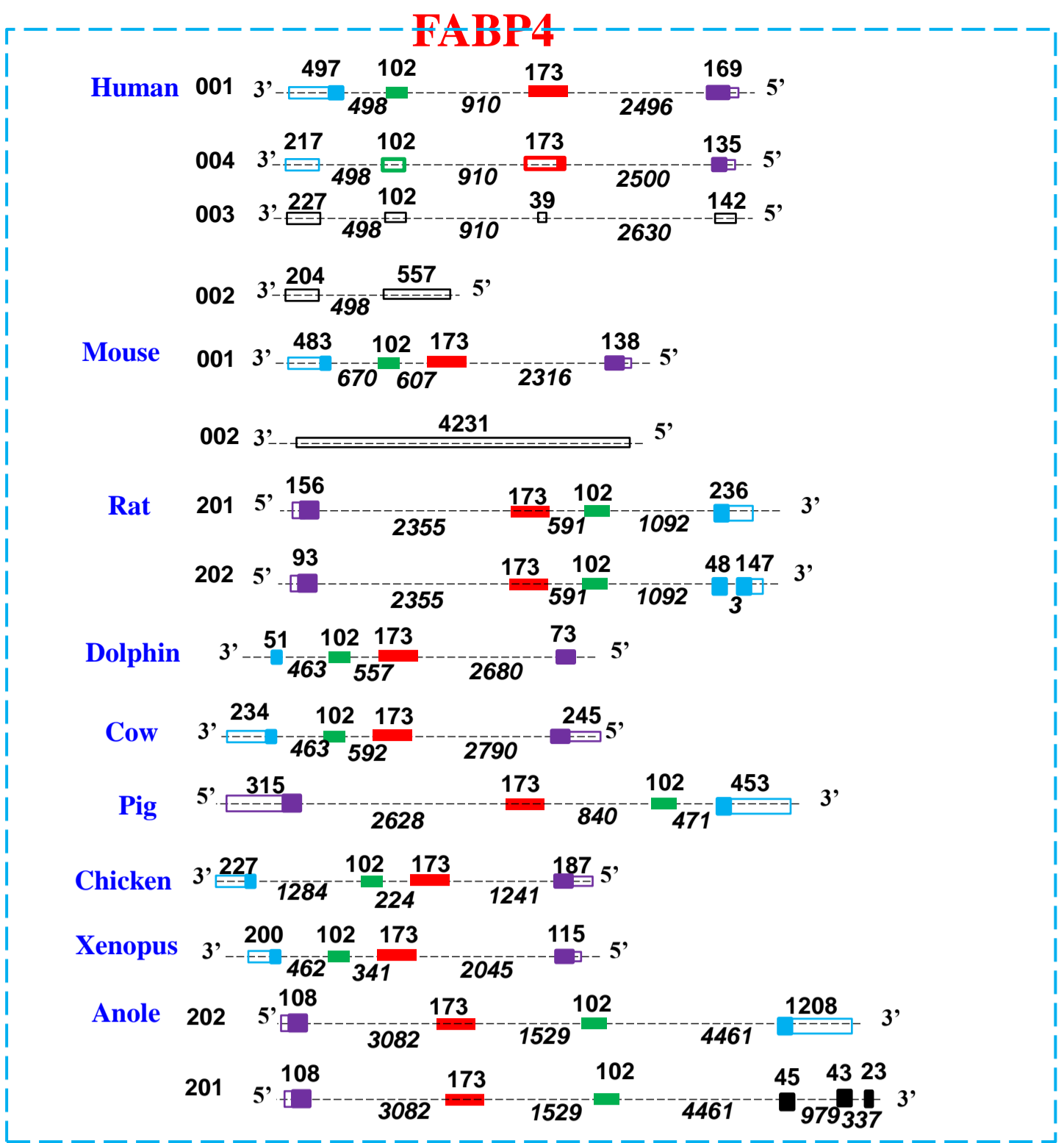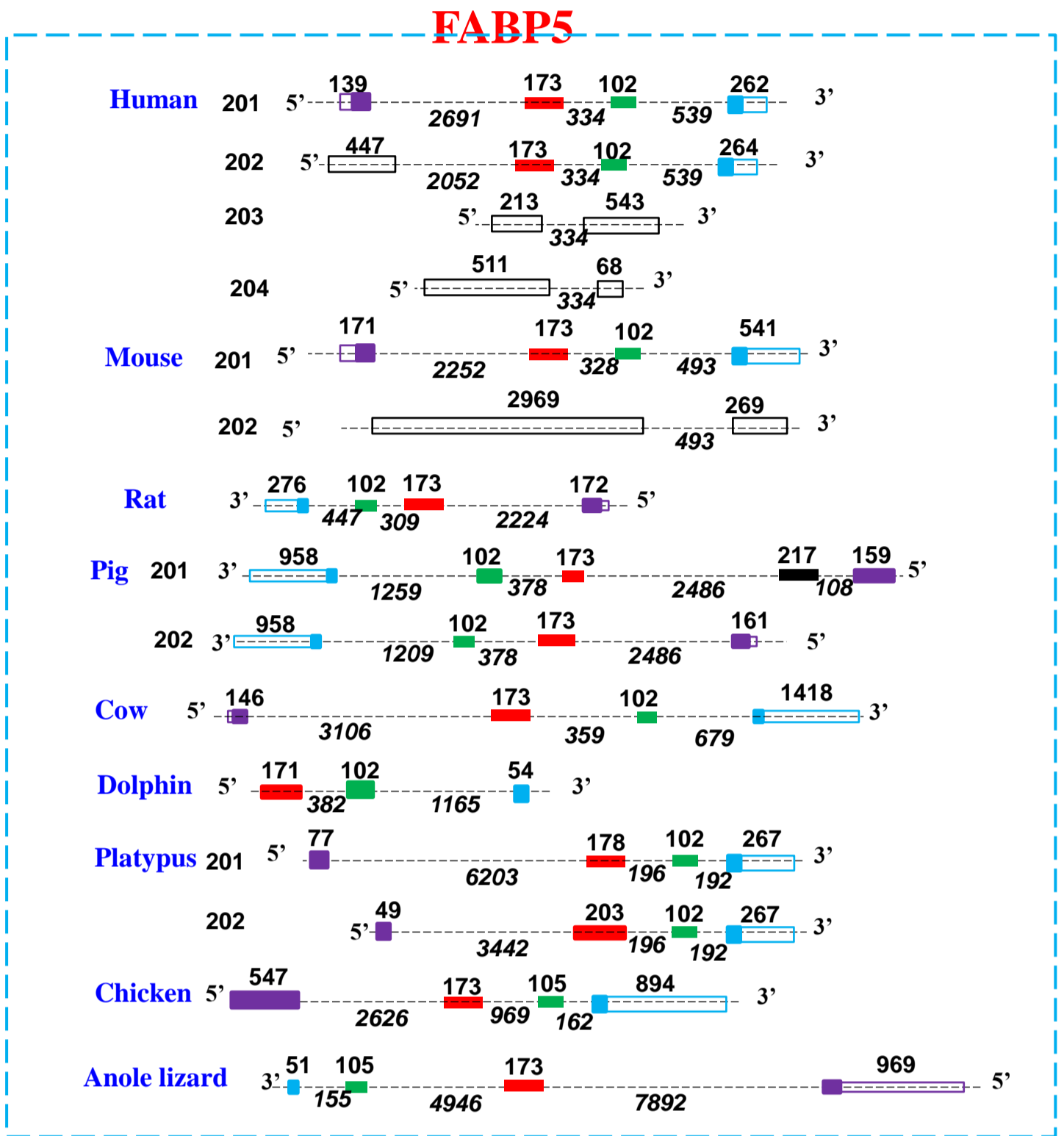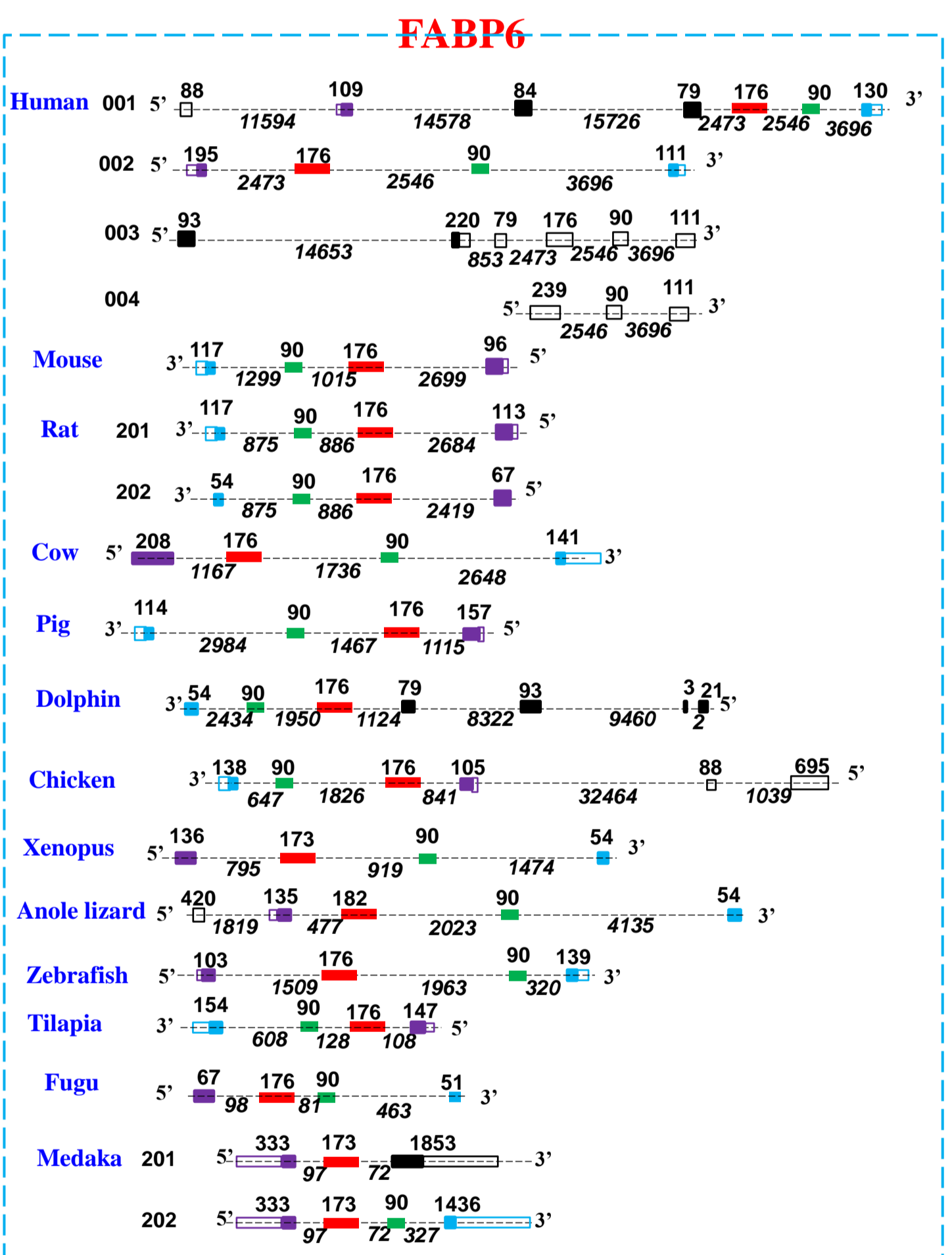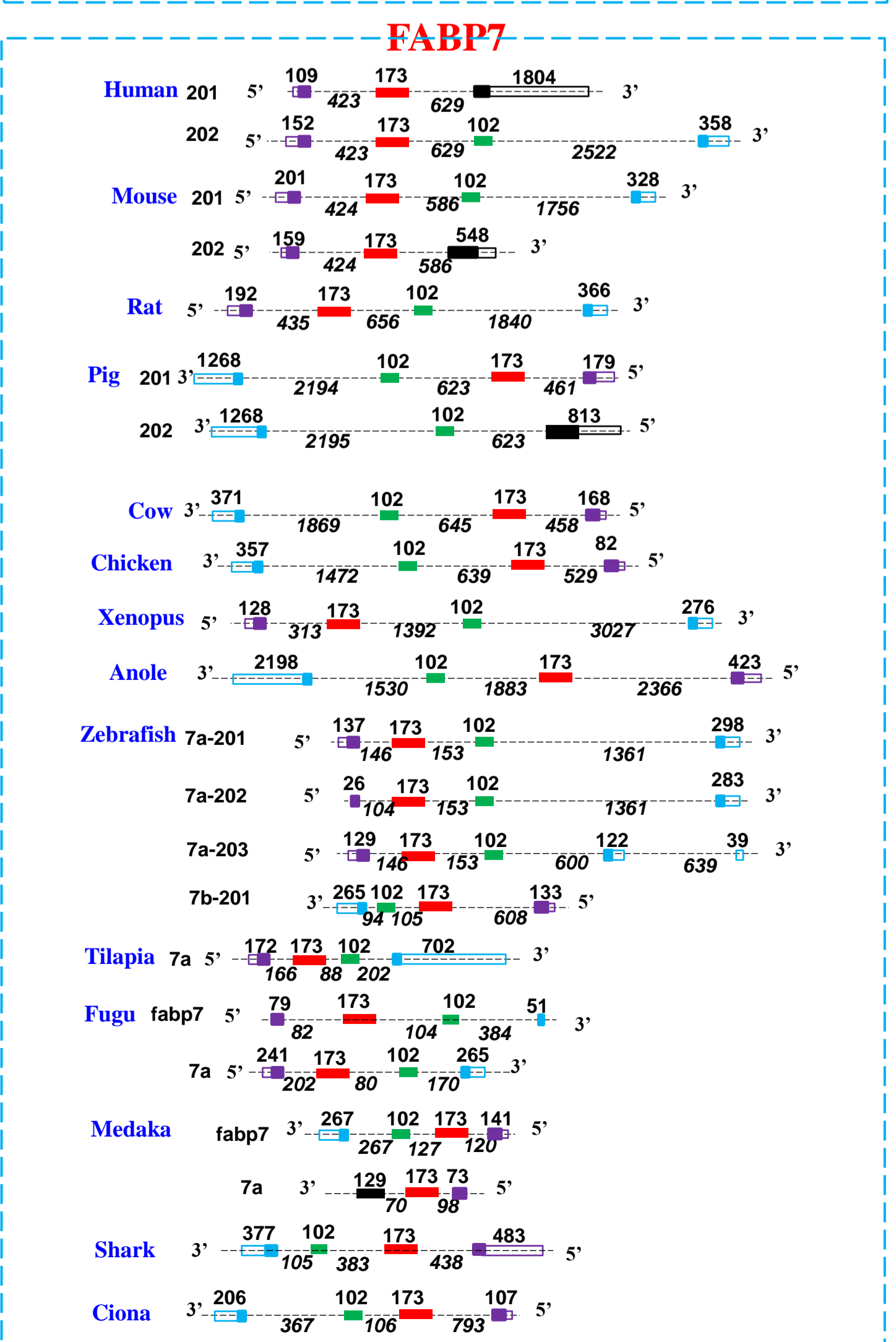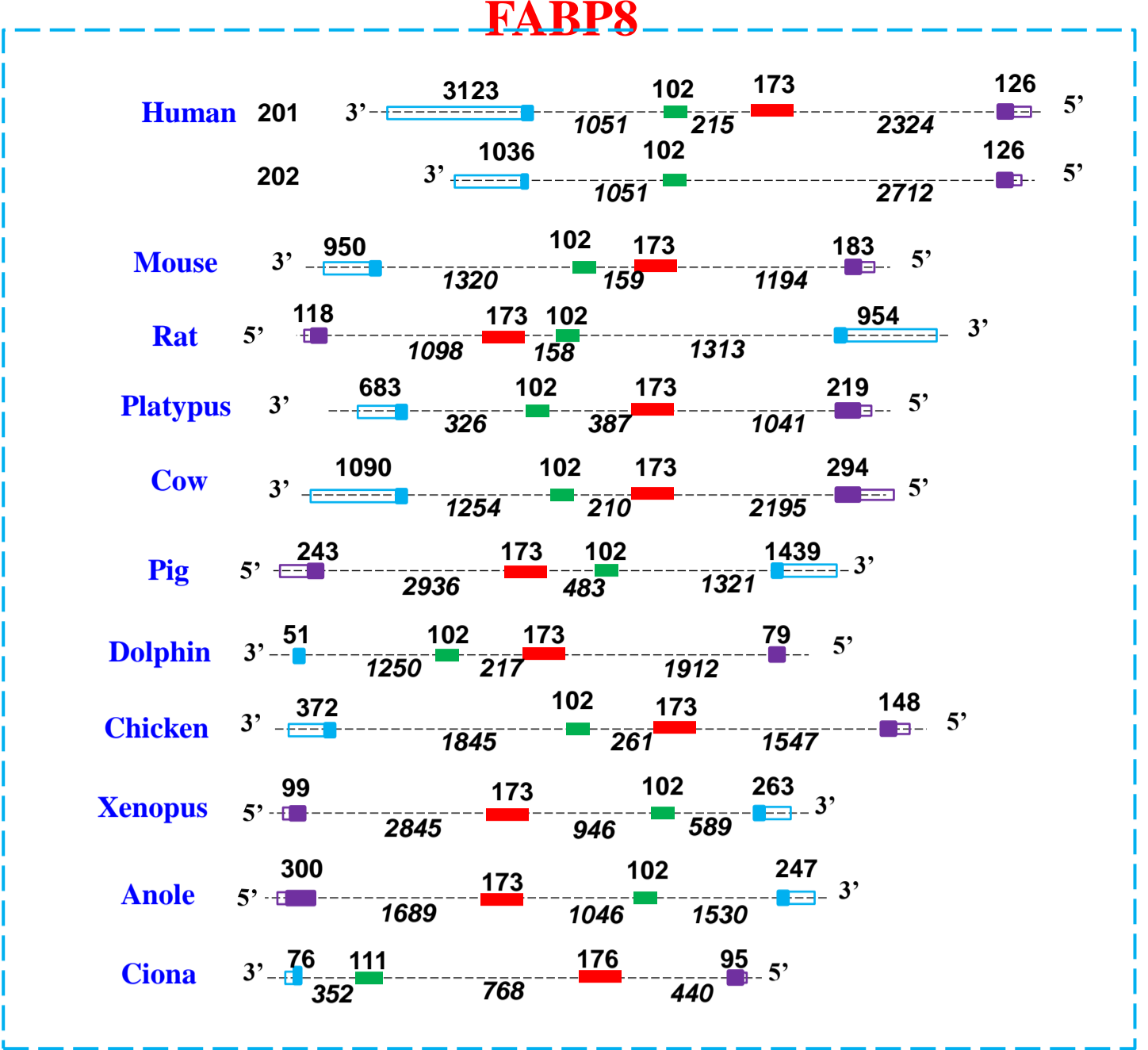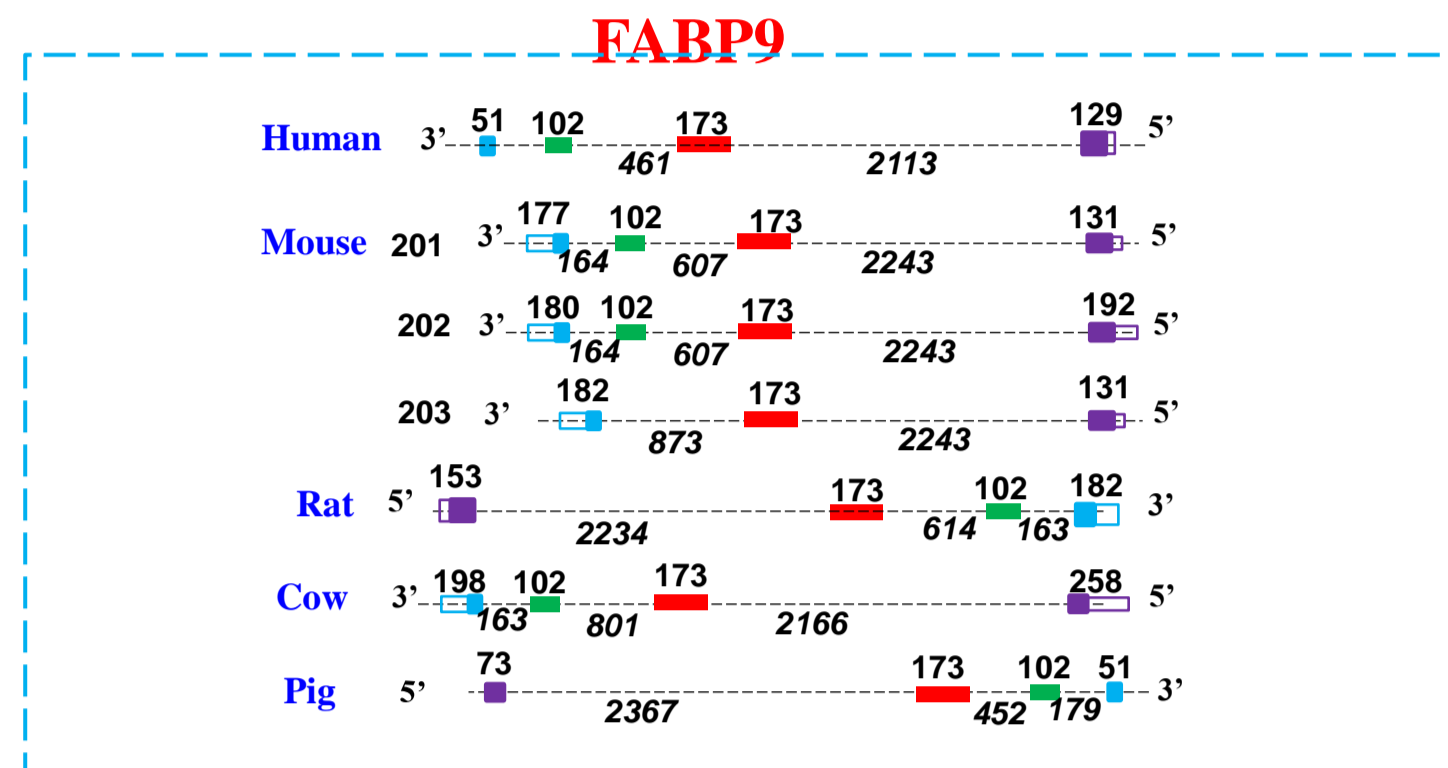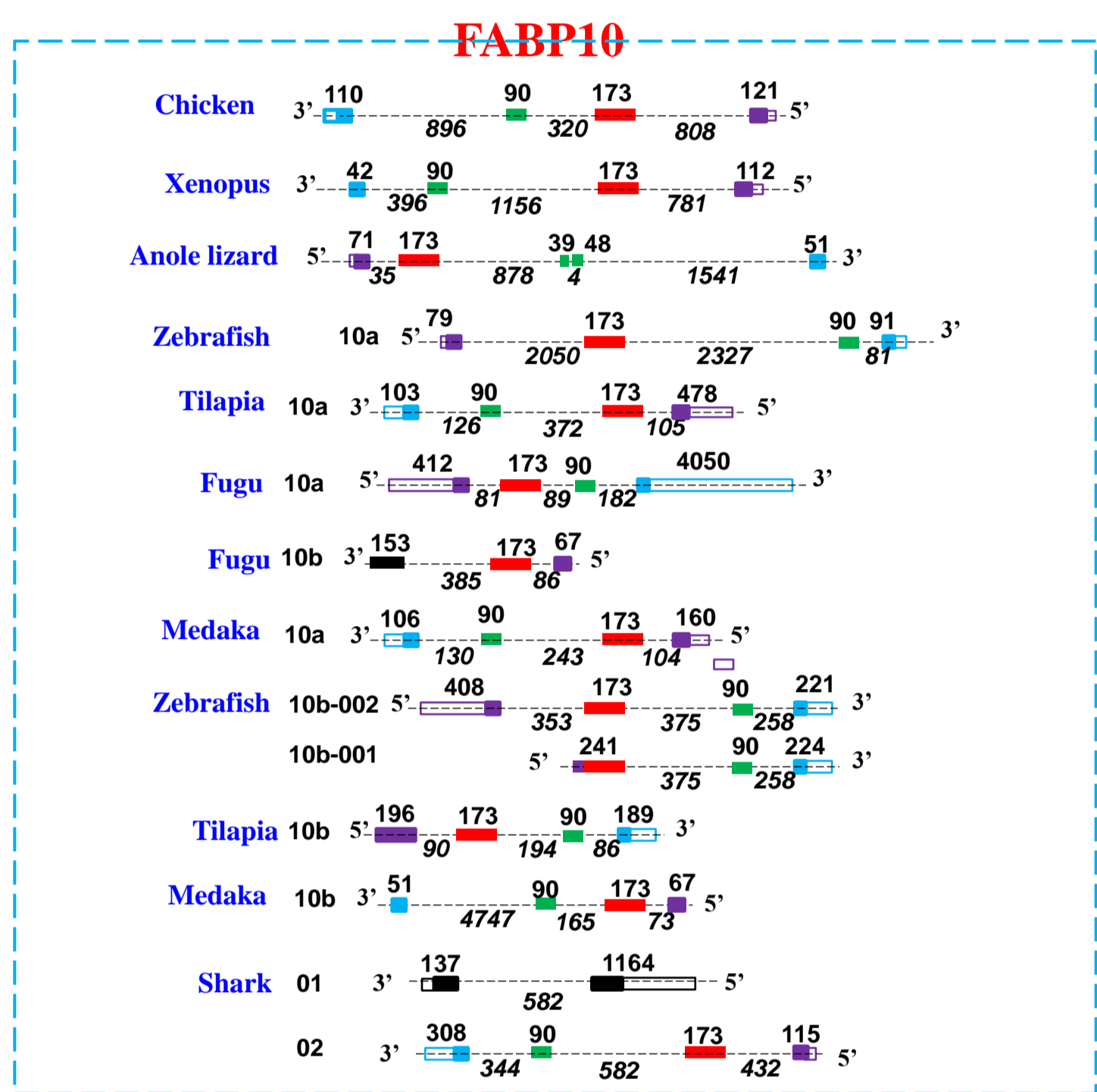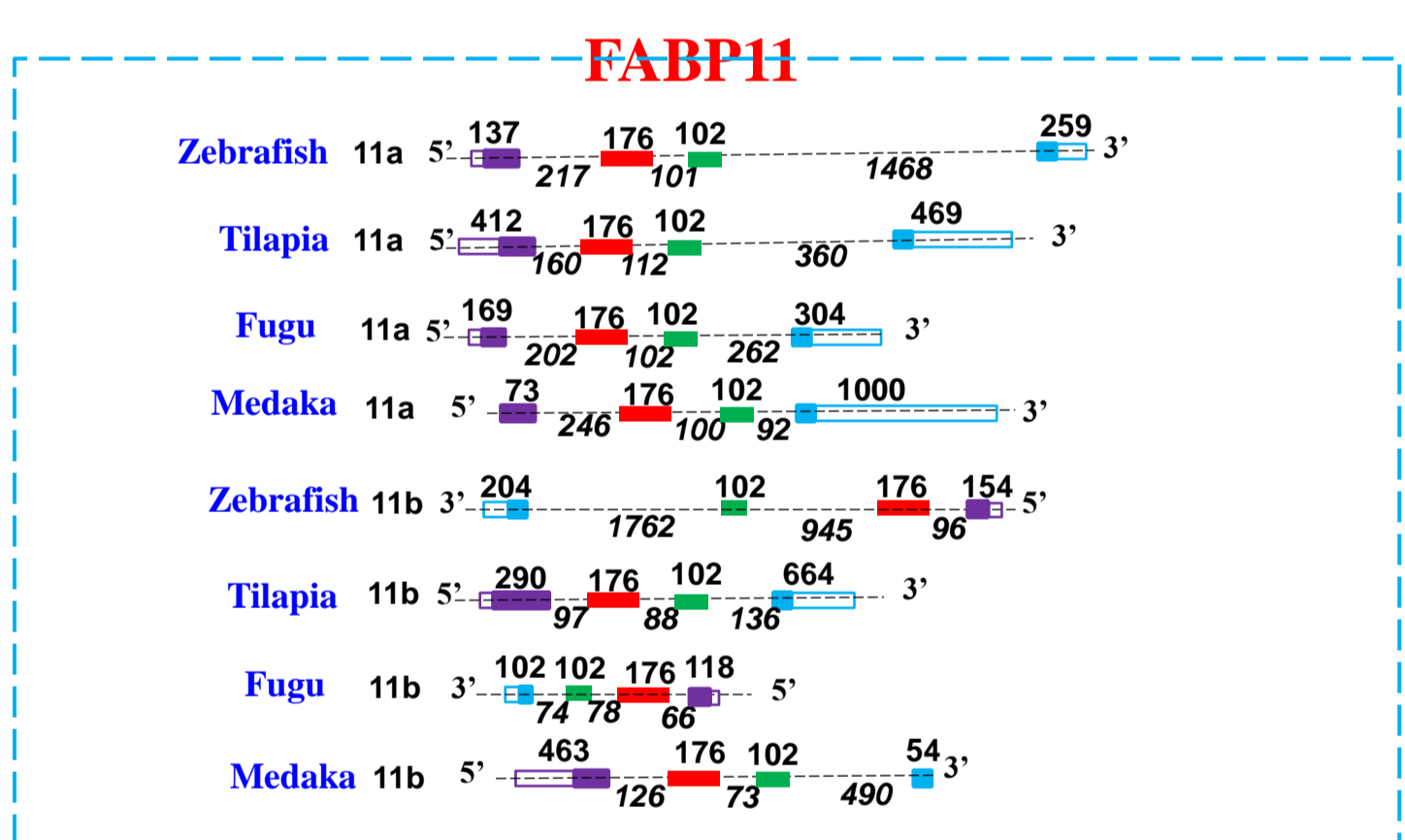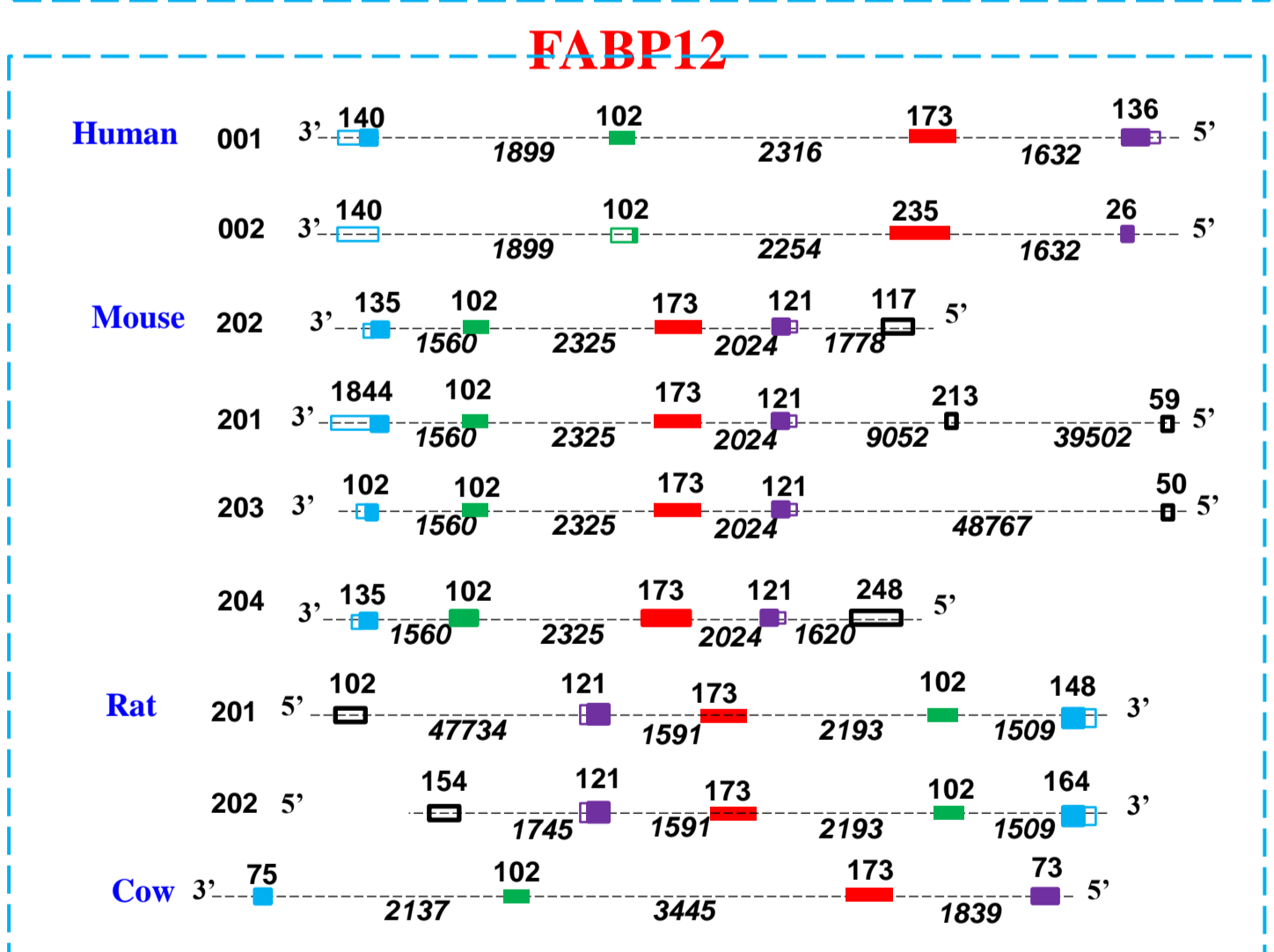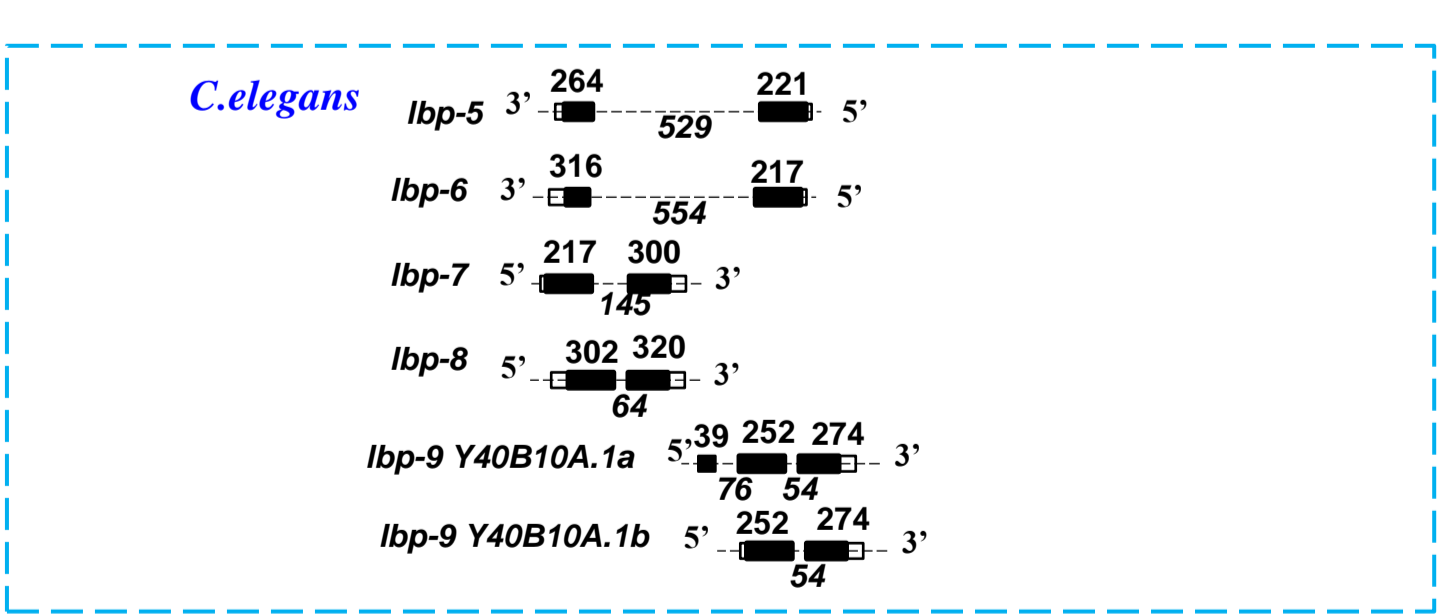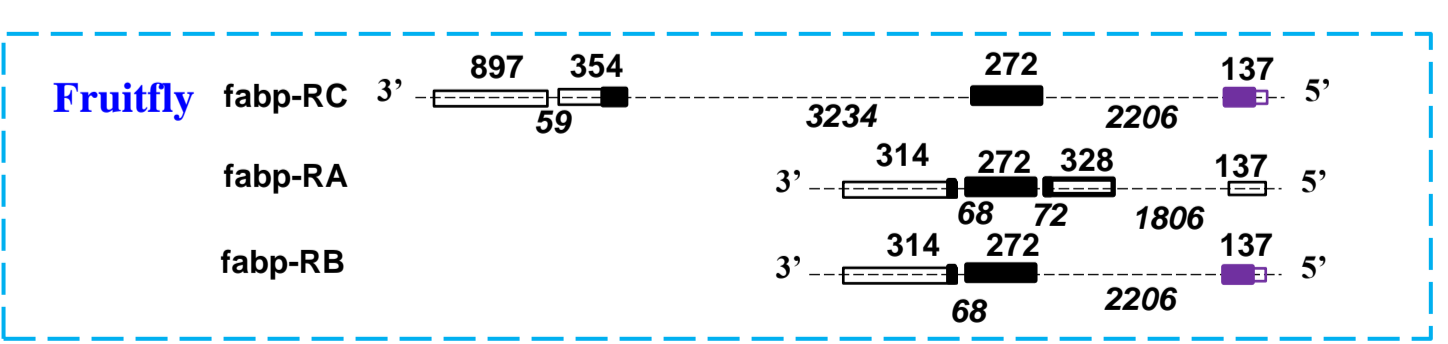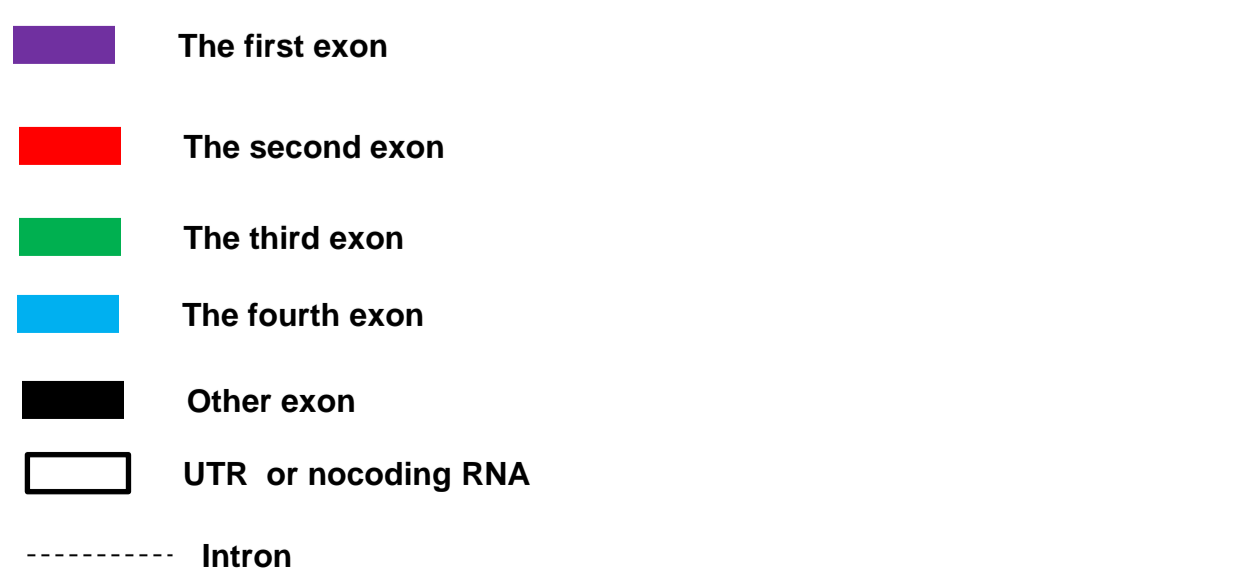

Supplement: Supplementary file 1 — Fig. S1. High‐resolution gene structure in PDF format. [file FEB4-10-861-s001.pdf]

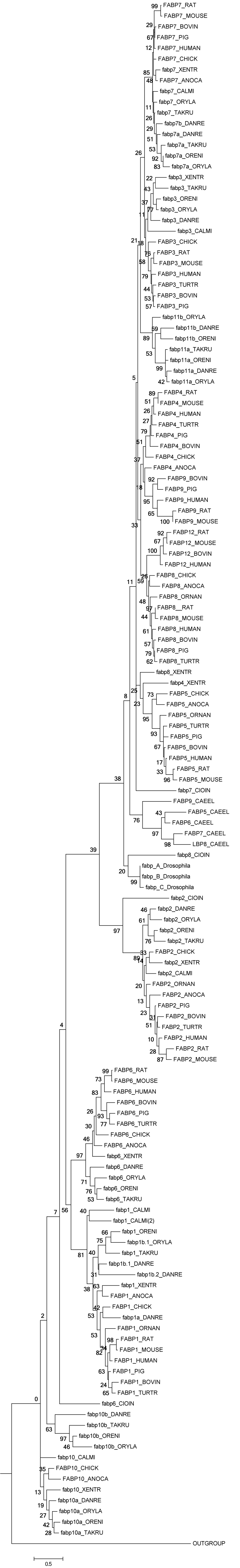

Supplement: Supplementary file 2 — Fig. S2. ML tree of FABP proteins. [file FEB4-10-861-s002.tif]
